# Predictive value of digital breast tomosynthesis based intratumoral-peritumoral radiomics and clinical features for benign-malignant breast microcalcifications

**DOI:** 10.1093/bjro/tzag020

**Published:** 2026-10-05

**Authors:** Junting Wei, Jie Li, Ruixin Pan, Yun Zhu, Zhizhen Gao

**Affiliations:** School of Medical Imaging, Bengbu Medical University, Bengbu 233030, China; Department of Radiology, The First Affiliated Hospital of Bengbu Medical University, Bengbu 233099, China; School of Medical Imaging, Bengbu Medical University, Bengbu 233030, China; Department of Radiology, The First Affiliated Hospital of Bengbu Medical University, Bengbu 233099, China; School of Medical Imaging, Bengbu Medical University, Bengbu 233030, China; Department of Radiology, The First Affiliated Hospital of Bengbu Medical University, Bengbu 233099, China; Department of Radiology, The First Affiliated Hospital of Bengbu Medical University, Bengbu 233099, China; School of Medical Imaging, Bengbu Medical University, Bengbu 233030, China

**Keywords:** microcalcifications, digital breast tomosynthesis, radiomics, nomogram, peritumoral

## Abstract

**Objective:**

To investigate the value of clinical and radiomics features in distinguishing benign from malignant breast microcalcifications and develop a noninvasive model to reduce unnecessary biopsies.

**Methods:**

This retrospective study analyzed 181 microcalcification lesions from 172 patients. Lesions were divided into training/validation sets (7:3). Manual volumes of interest were delineated on digital breast tomosynthesis images. Radiomics features were extracted from intratumoral and peritumoral regions. Among 9 machine learning classifiers, the optimal performer was selected to develop 3 radiomics models: intratumoral model (RM0), intratumoral and peritumoral 1 mm model (RM1), intratumoral and peritumoral 3 mm model (RM3). Independent clinical risk factors identified by univariate and multivariate logistic regression were integrated with the optimal radiomics model to build a nomogram model (NM). Model performance was evaluated using receiver operating characteristic (ROC) curves, calibration curves, decision curve analysis, and clinical impact curves.

**Results:**

RM0 performed best. Independent risk factors included: Breast Imaging Reporting and Data System (BI-RADS) category (*P* < .001), history of diabetes (*P* = .023), and personal history of breast cancer (*P* = .034). In the training cohort, the areas under the ROC curve (AUC) were 0.884 (clinical model), 0.728 (RM0), and 0.942 (NM). In the validation cohort, AUCs were 0.881, 0.695, and 0.790, respectively.

**Conclusions:**

Single clinical features showed potential predictive value for microcalcifications, while the integrated model might not demonstrate adequate discriminative power.

**Advances in knowledge:**

This study highlights the need for noninvasive differentiation of microcalcifications and suggests that while individual clinical factors have value, combined modeling requires further optimization.

## Introduction

According to 2022 data from the World Health Organization (WHO) International Agency for Research on Cancer (IARC), the global incidence of breast cancer is 11.6%, which has become the malignant tumor with the highest incidence among women worldwide.^1^ Currently, routine clinical screening primarily employs ultrasound, MRI, and mammography. Among these, mammography serves as a vital method for early breast cancer screening due to its high sensitivity in detecting breast calcifications.^2^^,^^3^ Breast microcalcifications are deposits of calcium salts in breast tissue with diameters ranging from 0.1 mm to 1.0 mm^4^ and mainly composed of calcium oxalate or hydroxyapatite.^5^ Reported positive predictive values for suspicious microcalcifications range widely from 15.9% to 90.6%.^6^ The clinical diagnosis is mainly confirmed by percutaneous biopsy or surgical excision, which may lead to unnecessary harm to some patients with benign microcalcifications. Conventional mammography can be hampered by overlapping glandular tissue, posing a risk of missed lesions, particularly in patients with dense breasts. Digital breast tomosynthesis (DBT) addresses this limitation by reconstructing 3D images from low-dose X-ray data acquired at multiple angles, enabling stratified visualization of breast structures.^7^ However, its diagnostic efficacy in distinguishing benign from malignant microcalcifications requires further validation. Radiomics facilitates the high-throughput extraction of quantitative features (texture, morphology, intensity) from medical images, thereby capturing biological information beyond traditional visual assessment.^8^ Current radiomics studies on breast microcalcifications have focused mainly on intratumoral feature analysis.^9^ As breast cancer is a highly heterogeneous tumor, the biological information embedded within the peritumoral microenvironment remains underexplored, and the diagnostic efficacy of microcalcifications is still unstable.

To address these challenges, this study integrates DBT with radiomics to systematically extract imaging features from both intratumoral and peritumoral regions of microcalcification lesions. These features are combined with clinical data, radiological characteristics, and laboratory indicators to develop a machine learning-based visual nomogram model (NM). The aim is to provide a noninvasive tool for distinguishing benign from malignant microcalcifications, potentially reducing unnecessary biopsies.

## Methods

### Study samples

Institutional Review Board approval was obtained, which granted a waiver of informed consent for this retrospective analysis. A total of 172 patients with pathologically confirmed breast microcalcifications were enrolled between April 2019 and June 2025 from a hospital. The sample comprised all consecutive eligible cases during this period, as no formal *a priori* sample size calculation was performed. All lesions were confirmed as benign or malignant by percutaneous biopsy or surgical resection, with histopathology used as the reference standard. The inclusion criteria were: (1) pre-procedural or preoperative DBT examination, (2) presence of radiographically evident microcalcifications on mammography, and (3) definitive histopathological diagnosis. Exclusion criteria were: (1) missing imaging data or poor image quality; (2) previous relevant treatment or invasive examination of the affected breast; (3) microcalcifications associated with surrounding masses, architectural distortion, or asymmetric densities (Figure 1).

**Figure 1:**
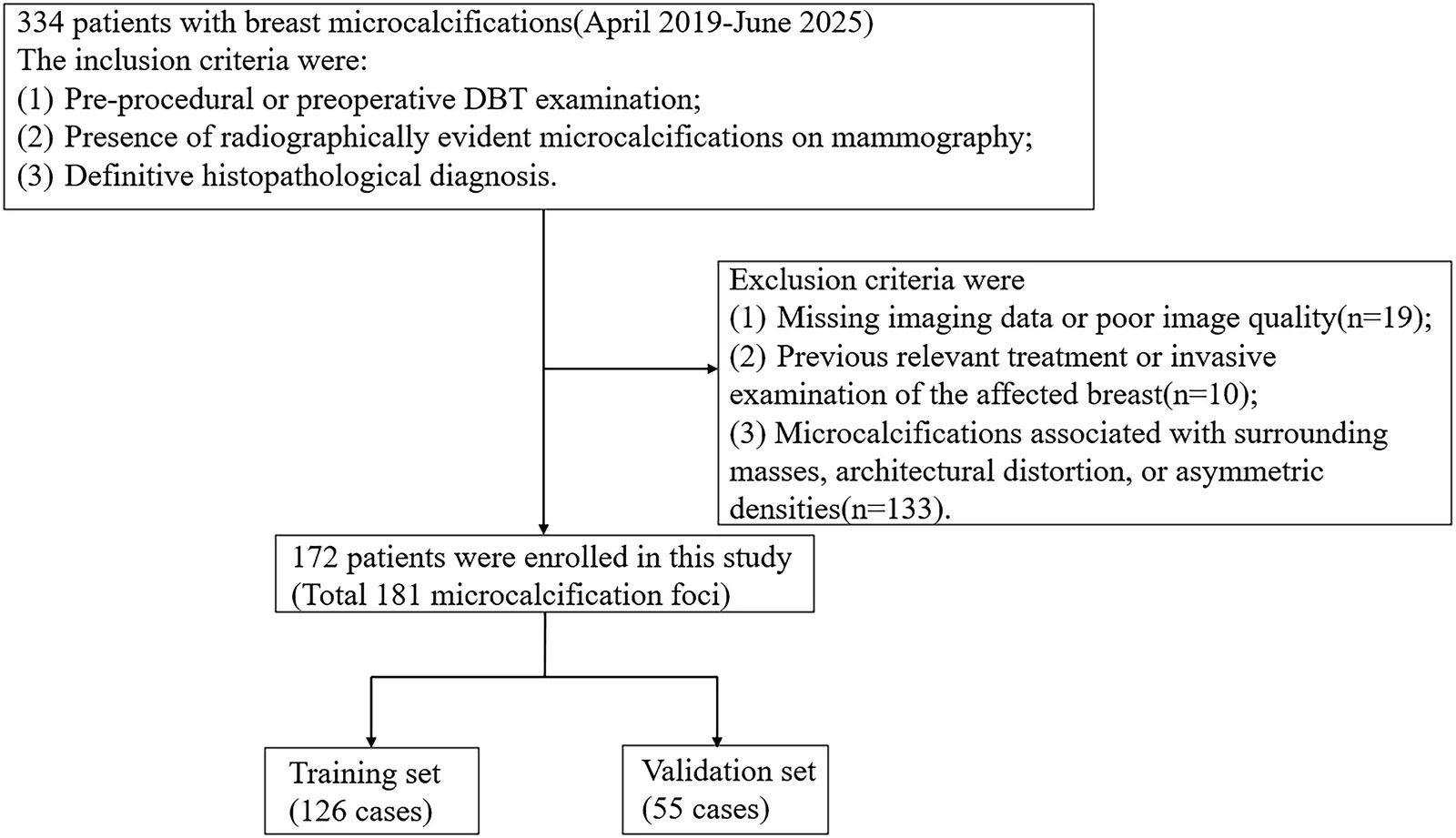
Patient screening flowchart based on inclusion/exclusion criteria.

### Imaging equipment and protocol

Imaging was acquired using a Siemens MAMMOMAT Inspiration full-field digital mammography system (Siemens Healthcare GmbH). Bilateral mammograms were acquired in the standard craniocaudal (CC) and mediolateral oblique (MLO) projections under “OPDOSE” mode conditions. For DBT acquisition, the X-ray tube was rotated through an arcuate trajectory of ±25°, obtaining low-dose projection images at 2° increments (25 total exposures). Thin-section images were subsequently reconstructed at 1 mm slice thickness using dedicated workstation software. All images were archived in Digital Imaging and Communications in Medicine (DICOM) format and uploaded to the institutional Picture Archiving and Communication System.

### Data analysis

#### Clinical and laboratory data

Clinical parameters and laboratory indices were obtained from electronic medical records via the hospital’s Radiology Information System. Clinical variables included age, lesion location, personal history of breast cancer, family history of breast cancer, reproductive history, menopausal status, and history of hypertension, diabetes, and coronary artery disease. The criteria for defining menopausal status, history of hypertension, diabetes, and coronary artery disease can be found in Supplementary Appendix E1. Laboratory indicators encompassed fasting serum levels of total cholesterol (TC, mmol/L), triglycerides (mmol/L), high-density lipoprotein cholesterol (mmol/L), and low-density lipoprotein cholesterol (LDL, mmol/L), measured within 1 week before percutaneous biopsy or surgical excision.

#### Imaging characteristics

The location of microcalcifications was recorded as upper outer quadrant or nonupper outer quadrant. Microcalcification lesions were stratified into the Fifth Edition Breast Imaging Reporting and Data System (BI-RADS) assessment categories 3, 4A-4C, and 5.^10^ The imaging characteristics were evaluated and analyzed by 2 radiologists, each with over 5 years of experience in diagnosing breast diseases. In cases of initial discrepancy in feature assessment, a final determination was made by a senior radiologist who held the professional title of associate chief physician or chief physician to reach consensus.

### Radiomics analysis

The acquired DBT images of the affected breast in CC and MLO projections were imported into the Darwin Intelligent Research Platform (http://www.yizhun-ai.com) in DICOM format. Two radiologists with at least 5 years of experience in breast imaging independently performed manual slice-by-slice delineation of volumes of interest (VOIs), covering all slices that contained microcalcifications. Both readers were blinded to the pathological results. Disagreements were resolved through joint discussion between the 2 radiologists or adjudication by a senior radiologist. The platform automatically generated expanded VOIs extending 1 and 3 mm peritumorally. Radiomics features, inclusive of both intratumoral and peritumoral characteristics, were subsequently extracted from these delineated VOIs using the platform. Prior to feature analysis, the intraclass correlation coefficient (ICC) was computed to assess the intra- and inter-observer reproducibility of radiomics features and features exhibiting an ICC ≥ 0.8 were retained. Details of the feature selection process are provided in Supplementary Appendix E2.

The optimal intratumoral features selected by the Least Absolute Shrinkage and Selection Operator (LASSO) regression were then input into 9 distinct machine learning classifiers: random forest (RF), support vector machine, logistic regression (LR), decision tree (DT), eXtreme Gradient Boosting (XGBoost), light gradient boosting machine, K-nearest neighbors, gradient boosting decision tree (GBDT), and adaptive boosting. The best-performing classifier was selected. It was then used to build combined intratumoral and peritumoral radiomics models for the 1 and 3 mm regions. The predictive performance of the three distinct radiomics models—intratumoral (RM0), intratumoral and peritumoral 1 mm (RM1), and intratumoral and peritumoral 3 mm (RM3)—was compared using the area under the receiver operating characteristic (AUC-ROC) curve. The model with the highest discriminative ability was subsequently used to compute a radiomics score (Rad-score) on the Darwin Intelligent Research Platform. This Rad-score was combined with independent clinical risk factors identified through univariate and multivariate analyses to build an NM that visualizes the contribution of each predictor.

### Statistical analysis

Missing values in clinical data were handled by imputing continuous variables with the mean and categorical variables with the mode. For continuous variables, the normality of distribution was assessed using the Shapiro-Wilk test. Data conforming to a normal distribution were presented as the mean ± SD and compared between 2 independent groups using the independent samples *t*-test. For data that violated the normality assumption, results were expressed as median (interquartile range, IQR), and the Wilcoxon rank-sum test was employed for group comparisons. Categorical variables were described as frequencies and percentages (*n*, %). Differences between groups for categorical data were analyzed using the Pearson’s Chi-square test or Fisher’s exact test, as appropriate. The radiomics features were selected using a sequential pipeline, comprising maximum-minimum normalization, optimal feature screening, maximum relevance minimum redundancy (mRMR) filtering, and LASSO regression. All statistical analyses were performed using R software (version 4.4.2; R Foundation for Statistical Computing) and SPSS (version 26.0; IBM Corp). A 2-sided *P* < .05 was considered statistically significant.

## Results

### Comparison of baseline characteristics and construction of the clinical model

This study included 172 patients with breast microcalcifications. Of these, 163 cases had a single focus and 9 cases had 2 foci, resulting in a total of 181 lesions (100 benign and 81 malignant) (Table S1). No significant differences in baseline clinical factors were found between the training and validation cohorts (all *P* > .05) (Table 1). Using the definitive pathological diagnosis as the reference standard, univariate and multivariate logistic regression analyses were performed. Features demonstrating a significance level of *P* < .1 in the univariate analysis such as age, BI-RADS category, personal history of breast cancer, history of diabetes, menopausal status, TC, and LDL were included in the multivariate logistic regression analysis. Three independent risk factors were identified: BI-RADS category (*P* < .001), personal history of breast cancer (*P* = .034), and history of diabetes (*P* = .023) (Table 2). These were used to build the clinical model. Figure 2 shows that the training set AUC of the clinical model was 0.884 (95% CI, 0.811-0.957) and the validation set AUC was 0.881 (95% CI, 0.739-1.000).

**Figure 2:**
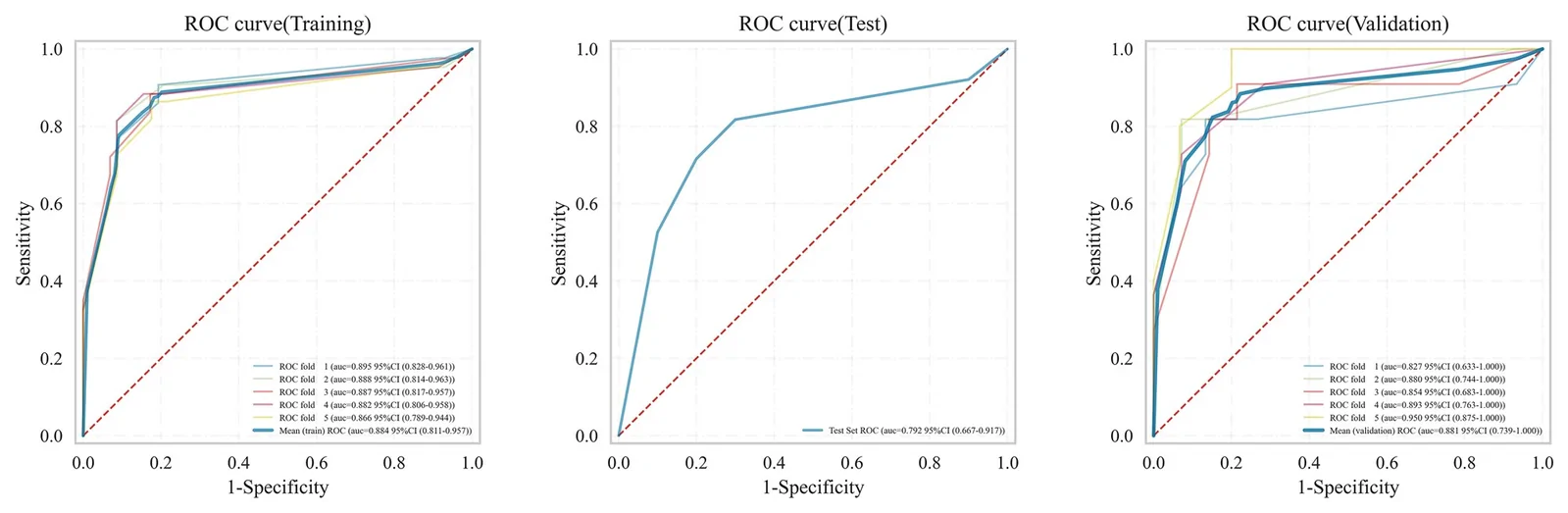
ROC curves of clinical model in training set, test set, and validation set.

**Table 1:** Comparison of basic clinical information between training and validation sets.

Variable	Group			*P*-value^a^
	Overall *n* = 181	Training set *n* = 126	Validation set *n* = 55	
**Age [Mean ± SD]**	48.12 ± 9.25	48.73 ± 9.77	46.71 ± 7.75	.341
**TC^b^ [Median (IQR)]**	4.63 (4.16, 5.00)	4.63 (4.13, 4.89)	4.63 (4.19, 5.31)	.401
**TG^c^ [Median (IQR)]**	1.24 (0.85, 1.55)	1.24 (0.86, 1.56)	1.15 (0.83, 1.41)	.665
**HDL^d^ [Median (IQR)]**	1.44 (1.24, 1.61)	1.41 (1.23, 1.60)	1.44 (1.25, 1.64)	.283
**LDL^e^ [Median (IQR)]**	2.52 (2.10, 2.93)	2.52 (2.07, 2.95)	2.52 (2.16, 2.84)	.948
**Lesion location [*n* (%)]**				.378
** Left**	103 (56.9%)	69 (54.8%)	34 (61.8%)	
** Right**	78 (43.1%)	57 (45.2%)	21 (38.2%)	
**Family history of breast cancer [*n* (%)]**				.667
** Yes**	7 (3.9%)	6 (4.8%)	1 (1.8%)	
** No**	174 (96.1%)	120 (95.2%)	54 (98.2%)	
**Reproductive history [*n* (%)]**				>.999
** Yes**	166 (91.7%)	115 (91.3%)	51 (92.7%)	
** No**	15 (8.3%)	11 (8.7%)	4 (7.3%)	
**Calcification location [*n* (%)]**				.835
** Upper outer quadrant**	127 (70.2%)	89 (70.6%)	38 (69.1%)	
** Non-upper outer quadrant**	54 (29.8%)	37 (29.4%)	17 (30.1%)	
**BI-RADS^f^ category [*n* (%)]**				.181
** 3**	12 (6.6%)	11 (8.7%)	1 (1.8%)	
** 4A**	85 (47.0%)	58 (46.1%)	27 (49.1%)	
** 4B**	23 (12.7%)	19 (15.1%)	4 (7.3%)	
** 4C**	44 (24.3%)	27 (21.4%)	17 (30.9%)	
** 5**	17 (9.4%)	11 (8.7%)	6 (10.9%)	
**Personal history of breast cancer [*n* (%)]**				.239
** Yes**	15 (8.3%)	8 (6.3%)	7 (12.7%)	
** No**	166 (91.7%)	118 (93.7%)	48 (87.3%)	
**History of diabetes [*n* (%)]**				>.999
** Yes**	9 (5.0%)	6 (4.8%)	3 (5.5%)	
** No**	172 (95.0%)	120 (95.2%)	52 (94.5%)	
**History of coronary artery disease [*n* (%)]**				.517
** Yes**	2 (1.1%)	1 (0.8%)	1 (1.8%)	
** No**	179 (98.9%)	125 (99.2%)	54 (98.2%)	
**Menopausal status [*n* (%)]**				.767
** Yes**	76 (42.0%)	52 (41.3%)	24 (43.6%)	
** No**	105 (58.0%)	74 (58.7%)	31 (56.4%)	
**History of hypertension [*n* (%)]**				.577
** Yes**	35 (19.3%)	23 (18.3%)	12 (21.8%)	
** No**	146 (80.7%)	103 (81.7%)	43 (78.2%)	

a *t*-test, Wilcoxon rank-sum test; Fisher’s exact test; Pearson’s Chi-square test.

b TC, total cholesterol.

c TG, triglycerides.

d HDL, high-density lipoprotein cholesterol.

e LDL, low-density lipoprotein cholesterol.

f BI-RADS, Breast Imaging Reporting and Data System.

**Table 2:** Univariate and multivariate logistic regression analysis table.

Characteristic	Descript	*P*	Univariable OR (95%CI)	*P*	Multivariable OR (95%CI)
**Age**	Mean ± SD	.003	1.05 (1.02-1.09)	.144	1.05 (0.98-1.12)
**TC^a^**	Median (IQR)	.056	1.46 (0.99-2.15)	.829	0.92 (0.44-1.95)
**TG^b^**	Median (IQR)	.236	1.21 (0.88-1.65)		
**HDL^c^**	Median (IQR)	.597	0.79 (0.34-1.87)		
**LDL^d^**	Median (IQR)	.079	1.54 (0.95-2.48)	.233	1.81 (0.68-4.78)
**Lesion location**	Left				
	Right	.239	0.70 (0.39-1.27)		
**Family history of breast cancer**	No				
	Yes	.918	0.92 (0.20-4.25)		
**Reproductive history**	No				
	Yes	.487	1.46 (0.50-4.20)		
**Calcification location**	Upper outer quadrant				
	Non-Upper outer quadrant	.302	0.71 (0.37-1.36)		
**BI-RADS^e^ category**	3				
	4A	.105	0.33 (0.09-1.26)		
	4B	.198	2.60 (0.61-11.15)		
	4C	.004	7.78 (1.91-31.73)		
	5	.005	15.00 (2.24-100.48)	<.001	4.48 (2.90-6.94)
**Personal history of breast cancer**	No				
	Yes	.028	3.77 (1.15-12.34)	.034	4.69 (1.12-19.64)
**History of diabetes**	No				
	Yes	.060	4.64 (0.94-22.96)	.023	10.45 (1.38-78.86)
**History of coronary artery disease**	No				
	Yes	.881	1.24 (0.08-20.10)		
**Menopausal status**	No				
	Yes	<.001	3.07 (1.66-5.66)	.612	0.74 (0.24-2.24)
**History of hypertension**	No				
	Yes	.209	1.61 (0.77-3.38)		

a TC, total cholesterol.

b TG, triglycerides.

c HDL, high-density lipoprotein cholesterol.

d LDL, low-density lipoprotein cholesterol.

e BI-RADS, Breast Imaging Reporting and Data System.

### Radiomics model construction

3D calcification regions were manually delineated on both CC and MLO projection images for each patient (Figure S1). A total of 3562, 7124, and 7124 radiomics features were initially extracted for intratumoral, intratumoral and peritumoral 1 mm, and intratumoral and peritumoral 3 mm regions, respectively. Results after ICC screening are shown in Supplementary Appendix E3. The remaining 9 (Table S2), 4 (including 2 intratumoral and 2 peritumoral features), and 8 (including 6 intratumoral and 2 peritumoral features) optimal radiomics features were identified (Figure S2). The 9 classifiers showed that the AUC of the training set ranged from 0.738 to 1.000 and the validation set AUC ranged from 0.474 to 0.708 (Figure 3). Four classifiers (XGBoost, RF, DT, and GBDT) showed clear signs of overfitting. In contrast, LR demonstrated the smallest discrepancy in performance, with a training set AUC of 0.738 (95% CI, 0.657-0.820) and a validation set AUC of 0.708 (95% CI, 0.535-0.881), indicating relatively stable performance. Consequently, LR was selected as the classifier for subsequent model construction. The optimal radiomics feature sets for each model were entered into the LR classifier, with stability assessed via 5-fold cross-validation. The AUC results for RM0, RM1, and RM3 in the training cohort were 0.728 (95% CI, 0.627-0.829), 0.668 (95% CI, 0.560-0.776), and 0.732 (95% CI, 0.632-0.832), respectively; corresponding validation cohort AUCs were 0.695 (95% CI, 0.482-0.905), 0.621 (95% CI, 0.385-0.856), and 0.691 (95% CI, 0.479-0.897) (Figure 4).

**Figure 3:**
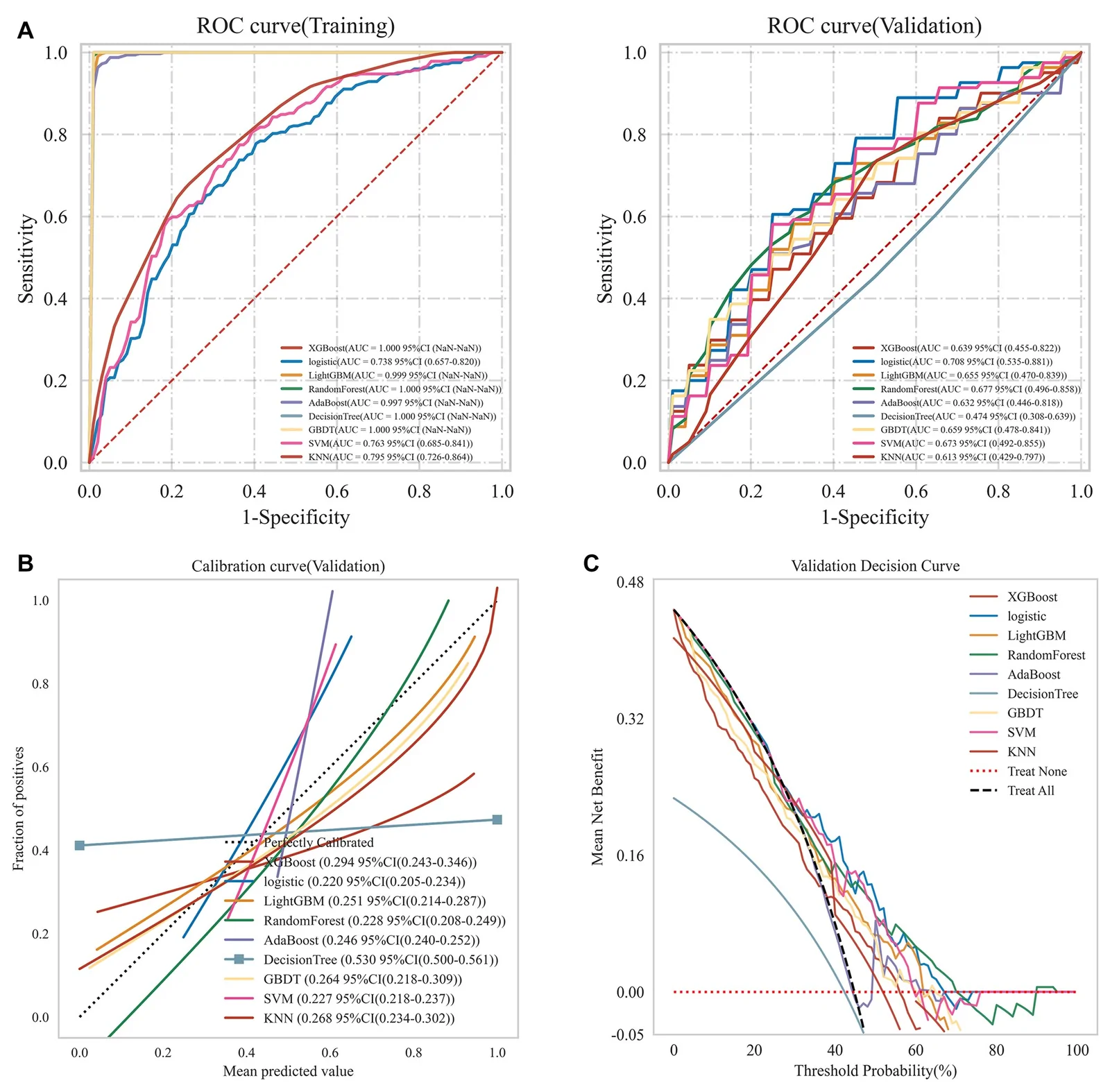
ROC curves for 9 machine classifiers in the training and validation sets (A); calibration curves for 9 machine classifiers in the validation set (B); decision curve analysis curves for 9 machine classifiers in the validation set (C).

**Figure 4:**
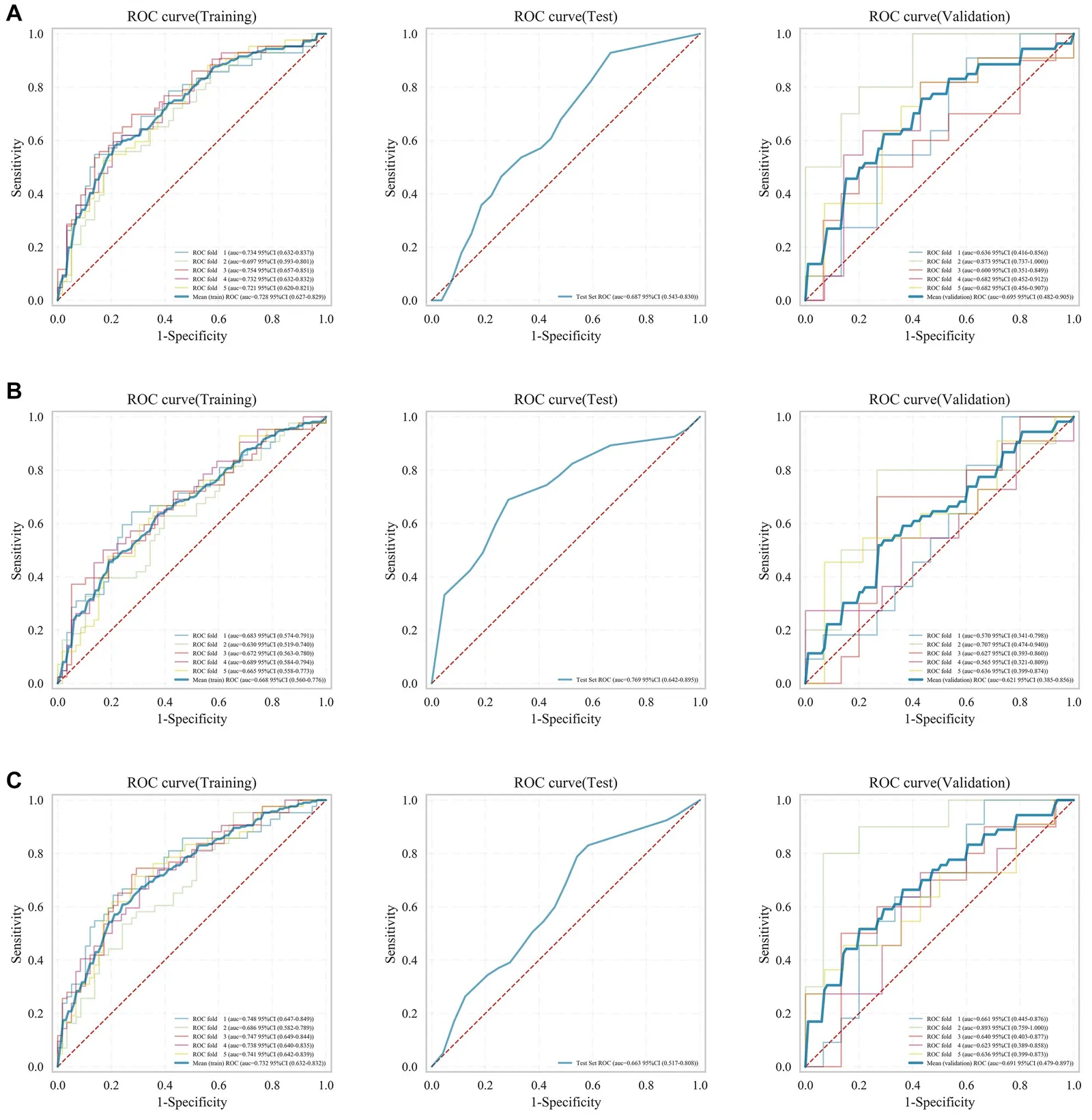
ROC curves of the radiomics models in the training set, test set, and validation set: RM0 (A); RM1 (B); RM3 (C).

### NM construction

RM0 showed an AUC similar to that of RM3. Because RM0 required less complex feature extraction, its Rad-score was chosen for integration with the 3 independent clinical risk factors identified earlier (BI-RADS category, personal history of breast cancer, and history of diabetes) to build an NM (Figure S3). The NM achieved a training set AUC of 0.942 (95% CI, 0.905-0.979) but dropped to 0.790 (95% CI, 0.667-0.912) in the test set (Figure 5A). This marked decline suggests overfitting. Figure 5B indicates a reasonable proximity of the predicted probability curve to the ideal line. However, the Brier scores (0.098 for the training set, 0.220 for the test set) confirmed significant overfitting and poor calibration in the test set. The decision curve analysis showed that compared to the strategies of treating none or treating all patients, RM0 yielded a higher net clinical benefit within a threshold probability range of 0.2-0.7, while the clinical model and NM provided greater net clinical benefit within a range of 0.2-0.9. Within the 0.5-0.8 probability threshold range, the net benefit of the clinical model surpassed that of NM. Within the 0.2-0.5 range, the net benefits of the 2 models were approximately equivalent (Figure 6A). The clinical impact curve results for the 3 models showed that the clinical model demonstrated higher concordance with the actual outcome curve over a wider range compared to the other 2 models. When the risk threshold exceeded 0.2, the predicted high-risk curve of the clinical model exhibited a smaller deviation from the actual outcome curve, suggesting superior clinical predictive performance for this model at higher thresholds (Figure 6B).

**Figure 5:**
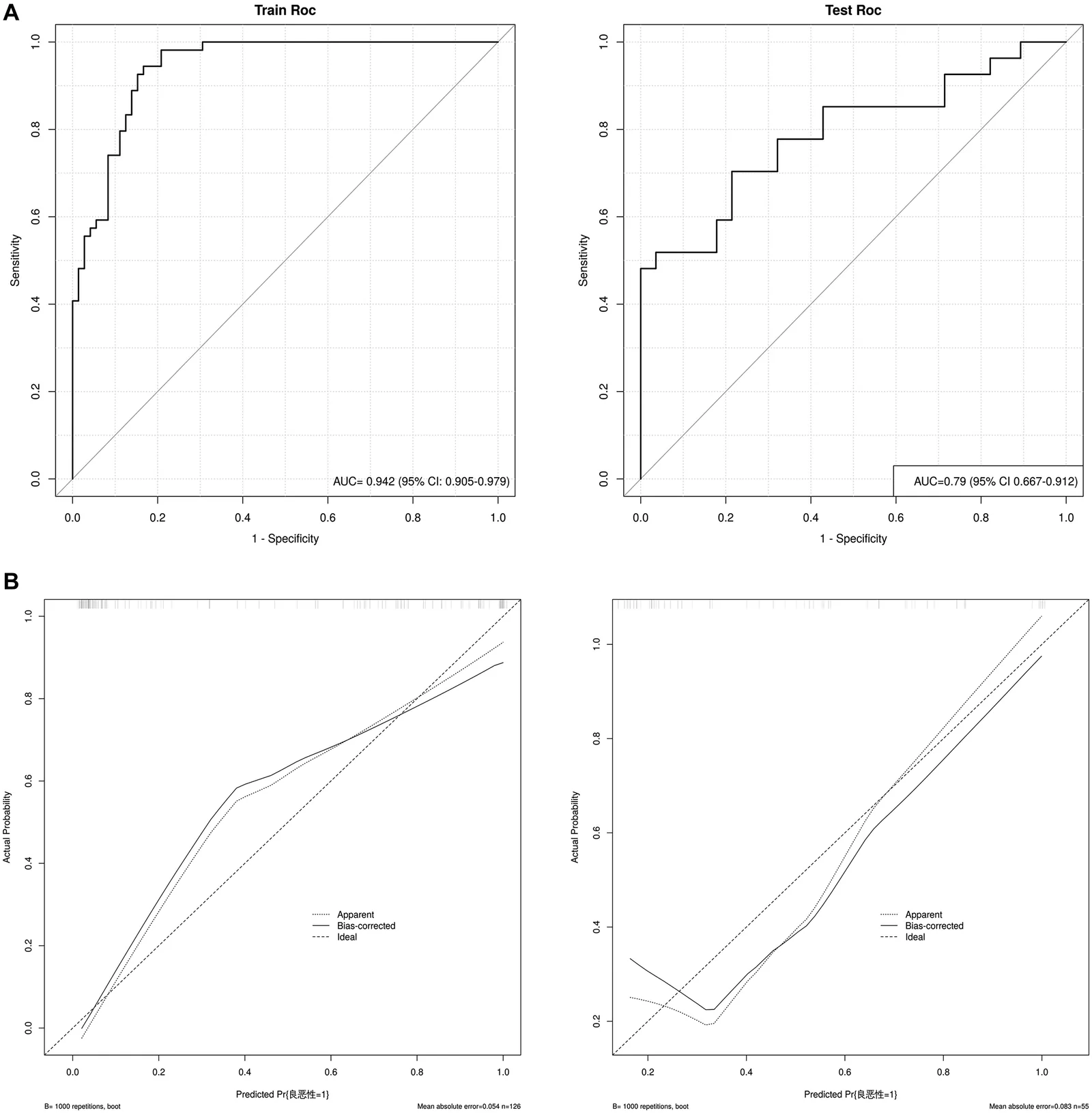
ROC curves for NM in the training and test sets (A); calibration curves for NM in the training and test sets (B).

**Figure 6:**
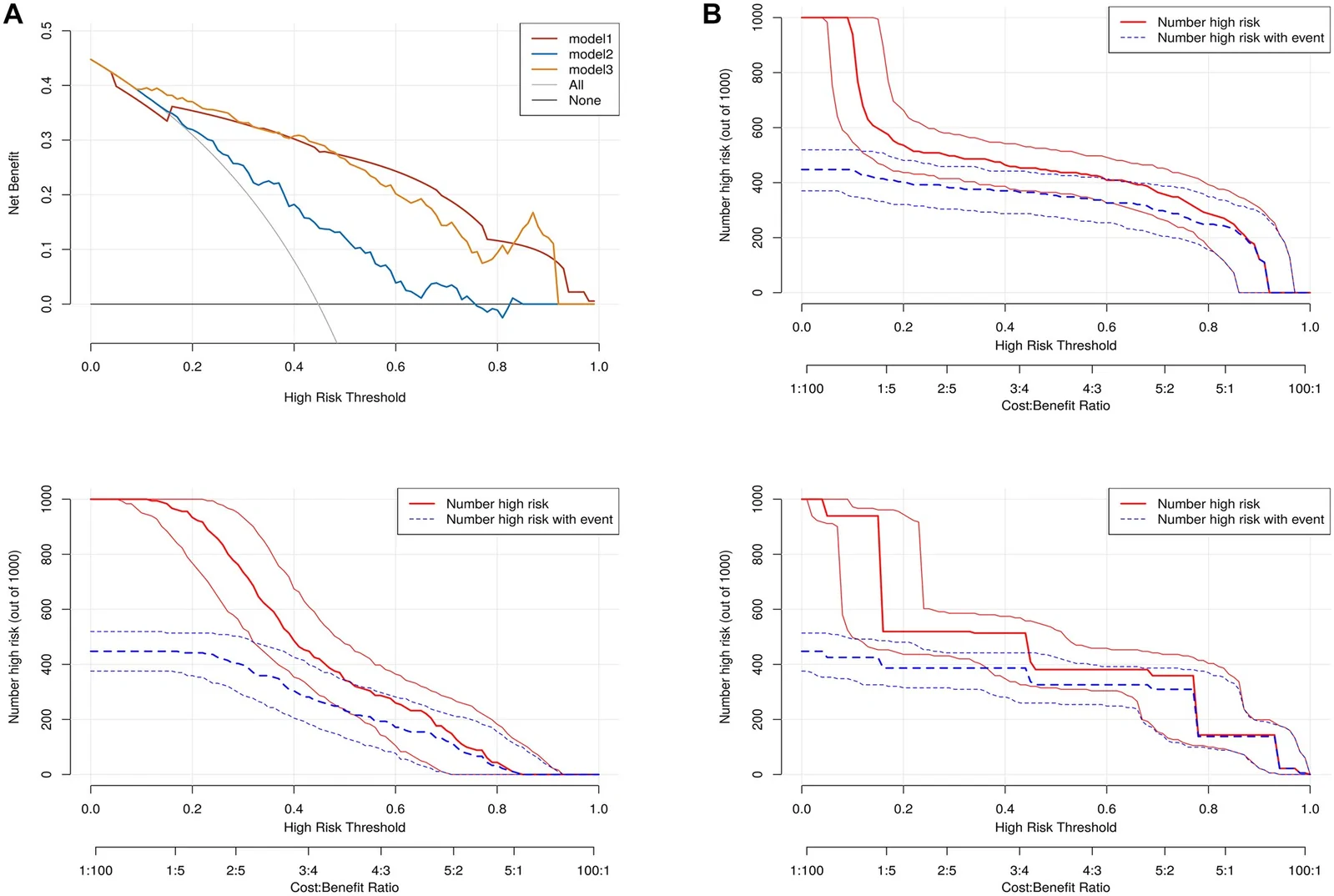
Decision curve analysis curves for 3 models, model 1: clinical model, model 2: RM0, model 3: NM (A); clinical impact curves for 3 models (B).

## Discussion

In this study, most patients underwent mammography voluntarily due to breast symptoms or increased health awareness, rather than a population-based screening program. More than 95% of patients in this study had dense breast tissue. In these women, clinicians often recommended additional DBT to further evaluate the malignant risk of lesions and obtain clearer 3D information for diagnosis. Although DBT is not yet widely used in population screening, its benefit for women with dense breasts has been recognized.^11^ Building upon this clinical context, this study innovatively extracted 3D radiomics features from DBT images and further extended the VOI to the 1-mm and 3-mm peritumoral regions to capture spatial heterogeneity beyond the microcalcification microenvironment. In addition, this study compared the diagnostic performance of 9 machine learning classifiers to identify the optimal algorithm for classifying breast microcalcifications. Our findings may inform future studies on feature extraction strategies and model selection.

Univariate and multivariate logistic regression analyses were performed on 15 clinical and imaging features, identifying 3 independent risk factors: BI-RADS category, personal history of breast cancer, and history of diabetes. BI-RADS is a standardized system for describing key mammographic findings, including masses, asymmetries, architectural distortion, calcifications, and associated features.^12^^,^^13^ Research on deep learning models integrating mammography and clinical factors showed good predictive performance (AUC = 0.905) in BI-RADS category 4 microcalcifications, while a model based solely on BI-RADS also showed relatively good predictive value (AUC = 0.802).^14^ A personal history of breast cancer is recognized as a high-risk factor, potentially attributable to residual tumor stem cells or micrometastases predisposing to contralateral breast cancer. Guidelines recommend annual breast screening for such patients, supplemented by MRI for enhanced sensitivity,^15–18^ with those having a history exceeding 10 years exhibiting significantly increased malignancy risk for suspicious calcifications.^19^ A history of diabetes may promote malignant calcification through metabolic dysregulation. Hyperglycemia contributes to endothelial damage, activating hypoxia-inducible factor driven tumor neovascularization, which facilitates cancer infiltration and calcification in necrotic zones. Concurrently, insulin resistance increases free fatty acids, triggering inflammation and accelerating stromal fibrosis, which may be associated with ductal carcinoma *in situ* (DCIS) progression.^20^^,^^21^ Diabetic breast cancer patients show higher mortality,^22^ underscoring metabolic factors’ critical role in malignant transformation.

Previous studies have shown the predictive value of peritumoral features in diverse tumor types,^23–25^ and evidence suggests that 3-mm peritumoral regions have prognostic value in breast cancer.^26^ Based on these findings, our study incorporated radiomics features from 1-mm to 3-mm peritumoral regions into our intratumoral model. However, this did not improve discriminative performance. This result may be explained by the pathogenesis of malignant microcalcifications and the specific characteristics of DBT imaging. At early stages, the peritumoral tissue may not yet exhibit pro-angiogenic or desmoplastic reactions, resulting in a lack of heterogeneous textural features associated with malignant progression in the peritumoral region. Hou et al found that the contribution of peritumoral microenvironment features in DCIS-associated calcifications to radiomics signatures was less than 5%, markedly differing from peritumoral features in invasive ductal carcinoma.^27^ Furthermore, the precise delineation of microcalcifications on tomographic slices may be limited by the inherent pixel resolution and reconstruction algorithms of DBT. Prior studies investigating DBT for calcification detection have not consistently yielded satisfactory results.^2^^,^^28^ Future studies should explore how DBT parameters, including reconstruction algorithms, slice thickness, and scanning angles, affect feature stability.

This study employed a full-slice delineation of VOIs, encompassing the spatial distribution of calcifications across all sectional slices. The resulting RM0 achieved a training AUC of 0.728, which was superior to that reported in previous studies that relied solely on 2D annotations of the central slice on DBT (AUC = 0.59)^29^ or features extracted from mammography images (AUC = 0.716).^9^ This may be attributed to several advantages. First, features such as the 3D Gray-Level Run-Length Matrix capture density gradient variations along the *Z*-axis. Second, 3D models reduce partial volume effects artifacts inherent to single-slice 2D delineation through anisotropic analysis. Nevertheless, the discriminative performance of all 3 studies (including ours) remains moderate. This may reflect the common limitations of radiomics studies with small sample size. The high number of radiomics features and the relatively small patient population restrict the upper limit of model performance. To our knowledge, no large-sample, multicenter radiomics studies based on DBT images for breast microcalcifications have been reported to date. Further validation is needed to improve model generalizability and clinical utility.

The clinical model outperformed RM0 in both the training set (AUC: 0.884 vs 0.728) and the validation set (AUC: 0.881 vs 0.695). This difference may be attributed to several factors. First, the study cohort had an unbalanced case distribution, with fewer malignant than benign lesions (malignant:benign = 4:5). In addition, more than half (54.3%) of the malignant cases were early-stage DCIS that did not show obvious architectural distortion on DBT images. Furthermore, current radiomics methods are more sensitive to textural heterogeneity in glandular tissue than to calcification-specific features. Because our VOIs included both microcalcifications and the surrounding background breast tissue, feature extraction may have detected a mixed signal from calcifications and adjacent glandular structures. To address these limitations, future studies should develop methods that extract novel imaging features with biological significance beyond current clinical factors, or leverage multi-modality deep learning for end-to-end, holistic decision optimization.

The NM showed a clear performance drop from the training set (AUC = 0.942) to the validation set (AUC = 0.790), along with a corresponding deterioration in calibration (Brier score = 0.220). This discrepancy indicates potential overfitting, suggesting that the model might have memorized training patterns but failed to generalize. This limitation may stem from the following mechanisms. First, radiomics and clinical features carried overlapping information, introducing multicollinearity that compromised model stability. Second, the current early feature concatenation approach did not balance the scaling or contribution weights between clinical and radiomics features. The Rad-score, despite its modest predictive power, may have diluted stronger clinical signals rather than complementing them. Future studies should explore advanced fusion strategies, such as weighted integration or deep learning-based multimodal fusion, to better harmonize feature interactions.

This study has several limitations. First, this was a single-center retrospective study with a small sample. No formal sample size calculation was performed, and external validation was not conducted, which limited the generalizability of our model. Second, the number of radiomics features was high relative to the small sample size, resulting in a risk of overfitting. The combined model showed poor calibration and provided no significant incremental clinical value. Third, our radiomics features captured both microcalcifications and background parenchymal signals, potentially reducing their specificity for microcalcifications and impacting diagnostic accuracy.

## Conclusion

Although the incorporation of peritumoral radiomics features did not lead to a further improvement in the diagnostic performance of the radiomics model, the intratumoral model established in this study demonstrated superior efficacy compared to previous studies. The predictive performance of the final combined model may be limited, highlighting the need for future studies to optimize radiomics feature extraction strategies to better capture microcalcification-specific characteristics. In current clinical practice, formally validated diagnostic tools should remain the first choice.

## Supplementary Material

tzag020_Supplementary_Data
