# Supplementary material for: Predictive value of digital breast tomosynthesis based intratumoral-peritumoral radiomics and clinical features for benign-malignant breast microcalcifications
Source: BJR Open. 2026 Oct 5;8(1):tzag020. doi: 10.1093/bjro/tzag020 (PMC13679928; doi:10.1093/bjro/tzag020)
Supplement: Supplementary file 1 [file tzag020_supplementary_data.zip › Supplementary material-changes (2).pdf]

Supplementary Material

Appendix E1

Menopausal status was classified as follows: women aged $\leqslant 50$ years with unavailable menstrual status data were classified as premenopausal; women aged >50 years or those with prior hysterectomy/bilateral oophorectomy resulting in amenorrhea were classified as postmenopausal.A history of hypertension was defined as a documented medical diagnosis of hypertension or ongoing receipt of antihypertensive medication; a history of diabetes was defined as a documented medical diagnosis of diabetes or current use of hypoglycemic agents/insulin; a history of coronary artery disease was defined as a diagnosis confirmed by coronary angiography or equivalent confirmatory diagnostic procedures.

Appendix E2

Radiomics Feature Selection

Maximum-minimum normalization was applied to eliminate scale differences among features, standardizing their values within a comparable range (-1 to 1), which was convenient for the subsequent processing of the model. Optimal feature screening was employed for dimensionality reduction: features most discriminative for benign and malignant calcifications were initially identified using the f_classif function; specifically, the top-performing 50% of intratumoral features and the top-performing 25% of features derived from both the intratumoral peritumoral 1mm and intratumoral peritumoral 3mm VOIs were retained. Subsequently, the Maximum Relevance Minimum Redundancy (mRMR) algorithm selected a subset of 50 features demonstrating maximal relevance to calcification malignancy status coupled with minimal mutual redundancy. Finally, the Least Absolute Shrinkage and Selection Operator (LASSO) regression with ten-fold cross-validation was utilized to tune the appropriate regularization strength parameter λ, thereby refining the selection to identify the features most significantly contributory to discriminating benign from malignant calcifications.

Appendix E3

After screening with ICC ≥ 0.8, the number of remaining features was as follows: intratumoral region: 2529 highly reproducible features were retained from the initial 3562 features; intratumoral + peritumoral 1 mm region: about 4631 highly reproducible features were retained from the initial 7124 features; intratumoral + peritumoral 3 mm region: about 4773 highly reproducible features were retained from the initial 7124 features.

Table S1：Pathologic types and composition ratio of 181 lesions

Nature of the lesion	Number of lesions	Pathological type	Number of detected cases	Percentage composition（%）
**Benign lesions**	100	Adenosis of the breast	65	65.00
		Fibroadenoma	14	14.00
		Intraductal papilloma	9	9.00
		Sclerosing adenosis	3	3.00
		Ductal epithelial hyperplasia	7	7.00
		Inflammatory lesions	1	1.00
		Mammary duct ectasia	1	1.00
**Malignant lesions**	81	Ductal carcinoma in situ	43	53.09
		Invasive carcinoma	30	37.04
		Mucinous carcinoma	3	3.70
		Lobular carcinoma in situ	1	1.23
		Ductal carcinoma in situ with focal microinvasion	4	4.94

Table S2：Optimal Intratumoral Radiomics Features and Their Coefficients with the Minimum Mean Squared Error.

Name	Minimum Mean Square Error
lbp2D_firstorder_10Percentile_MLO_intratumoral	-0.073
exponential_glszm_LowGrayLevelZoneEmphasis_CC_intratumoral	-0.142
lbp3Dk_glszm_SmallAreaEmphasis_CC_intratumoral	1.282
waveletLHH_glszm_SizeZoneNonUniformityNormalized_CC_intratumoral	1.239
waveletHLL_gldm_LargeDependenceLowGrayLevelEmphasis_CC_intratumoral	0.134
lbp3Dk_firstorder_10Percentile_MLO_intratumoral	0.654
lbp3Dk_firstorder_Skewness_MLO_intratumoral	0.194
waveletLLH_ngtdm_Busyness_MLO_intratumoral	0.731
waveletLHH_glcm_Correlation_MLO_intratumoral	0.572

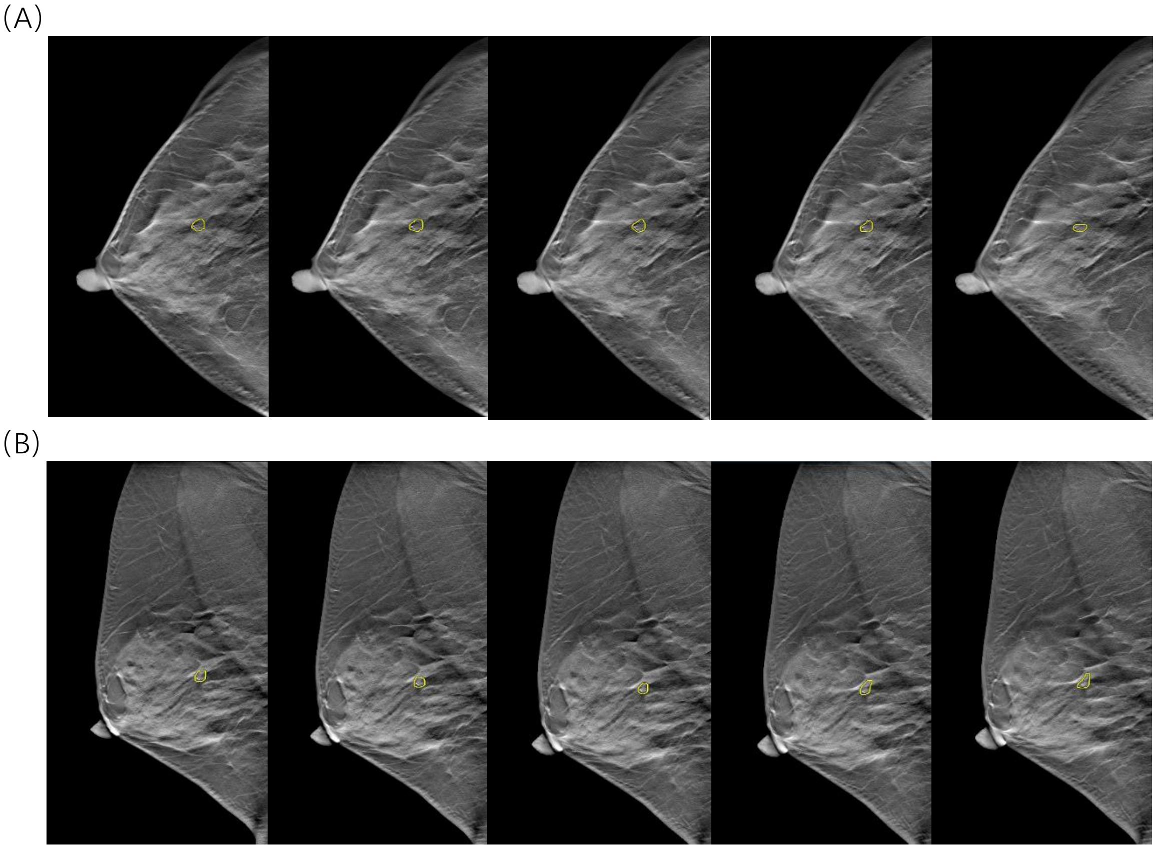

Figure S1: Partial calcification level outlined on DBT image; CC (A); MLO (B)

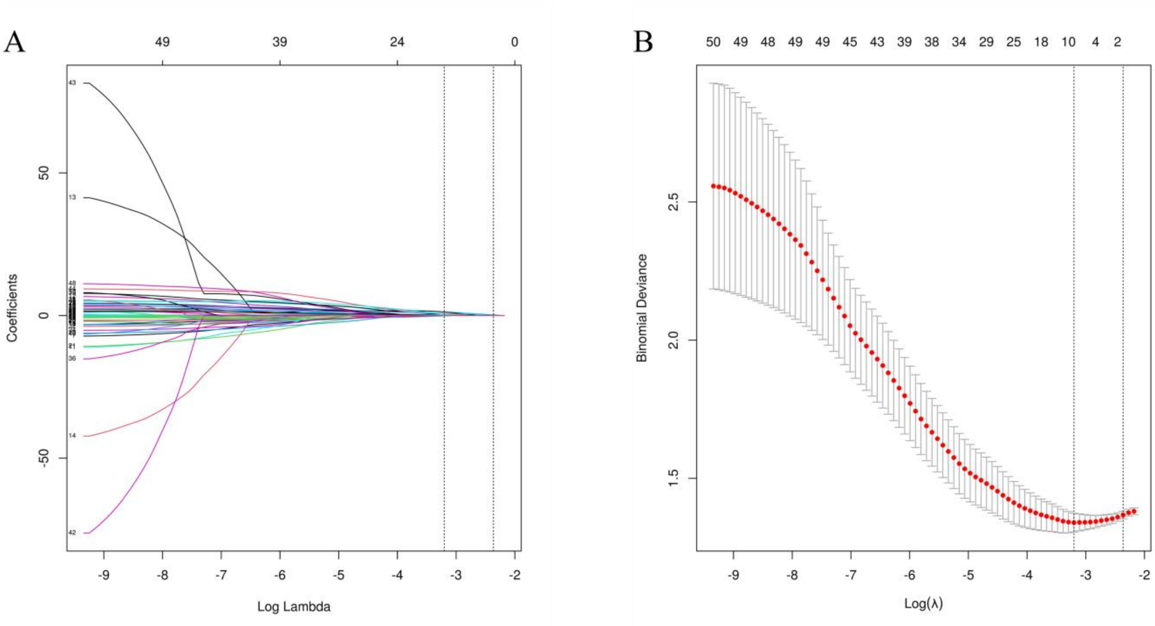

Figure S2: LASSO regression screening image histology characterization plot;Coefficient path plot (A); Cross-validation error plot (B).

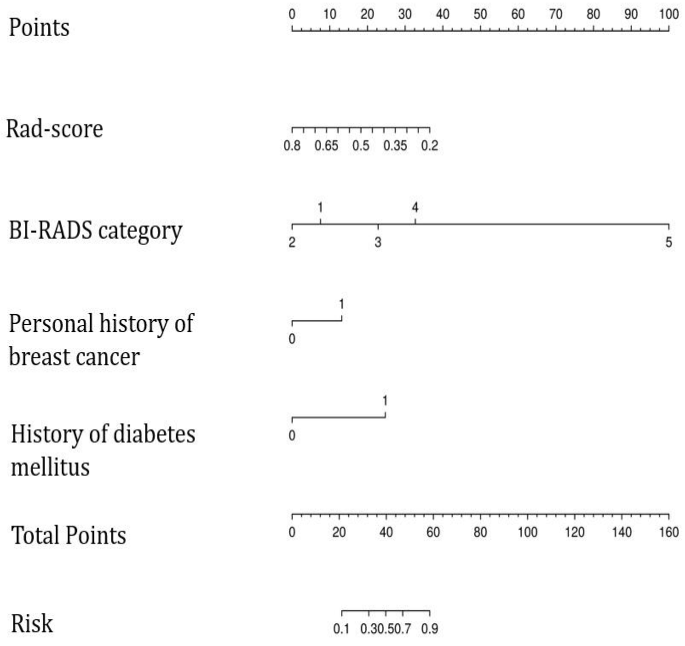

Figure S3: Nomogram for predicting the benign and malignant of breast microcalcifications.
