# Supplementary material for: Predictive value of digital breast tomosynthesis based intratumoral-peritumoral radiomics and clinical features for benign-malignant breast microcalcifications
Source: BJR Open. 2026 Oct 5;8(1):tzag020. doi: 10.1093/bjro/tzag020 (PMC13679928; doi:10.1093/bjro/tzag020)
Supplement: Supplementary file 1 [file tzag020_supplementary_data.zip › Supplementary material-table.docx]

Table S1：Pathologic types and composition ratio of 181 lesions

| **Nature of the lesion** | **Number of lesions** | **Pathological type** | **Number of detected cases** | **Percentage composition（%）** |
| --- | --- | --- | --- | --- |
| **Benign lesions** | 100 | Adenosis of the breast | 65 | 65.00 |
|  |  | Fibroadenoma | 14 | 14.00 |
|  |  | Intraductal papilloma | 9 | 9.00 |
|  |  | Sclerosing adenosis | 3 | 3.00 |
|  |  | Ductal epithelial hyperplasia | 7 | 7.00 |
|  |  | Inflammatory lesions | 1 | 1.00 |
|  |  | Mammary duct ectasia | 1 | 1.00 |
| **Malignant lesions** | 81 | Ductal carcinoma in situ | 43 | 53.09 |
|  |  | Invasive carcinoma | 30 | 37.04 |
|  |  | Mucinous carcinoma | 3 | 3.70 |
|  |  | Lobular carcinoma in situ | 1 | 1.23 |
|  |  | Ductal carcinoma in situ with focal microinvasion | 4 | 4.94 |

| **Name** | **Minimum Mean Square Error** |
| --- | --- |
| lbp2D_firstorder_10Percentile_MLO_intratumoral | -0.073 |
| exponential_glszm_LowGrayLevelZoneEmphasis_CC_intratumoral | -0.142 |
| lbp3Dk_glszm_SmallAreaEmphasis_CC_intratumoral | 1.282 |
| waveletLHH_glszm_SizeZoneNonUniformityNormalized_CC_intratumoral | 1.239 |
| waveletHLL_gldm_LargeDependenceLowGrayLevelEmphasis_CC_intratumoral | 0.134 |
| lbp3Dk_firstorder_10Percentile_MLO_intratumoral | 0.654 |
| lbp3Dk_firstorder_Skewness_MLO_intratumoral | 0.194 |
| waveletLLH_ngtdm_Busyness_MLO_intratumoral | 0.731 |
| waveletLHH_glcm_Correlation_MLO_intratumoral | 0.572 |

Table S2：Optimal Intratumoral Radiomics Features and Their Coefficients with the Minimum Mean Squared Error.
